# Temporal composition of the cervicovaginal microbiome associates with hrHPV infection outcomes in a longitudinal study

**DOI:** 10.1186/s12879-024-09455-1

**Published:** 2024-06-03

**Authors:** Mariano A. Molina, William P. J. Leenders, Martijn A. Huynen, Willem J. G. Melchers, Karolina M. Andralojc

**Affiliations:** 1 Department of Medical Microbiology, Radboud University Medical Center, Nijmegen, 6500 HB The Netherlands; 2 Department of Medical Microbiology, Radboud Institute for Molecular Life Sciences, Nijmegen, The Netherlands; 3 Predica Diagnostics, Toernooiveld 1, Nijmegen, 6525 ED The Netherlands; 4 Center for Molecular and Biomolecular Informatics, Radboud University Medical Center, Nijmegen, 6525 GA The Netherlands

**Keywords:** CVM, hrHPV, CSTs, Microbiome, Longitudinal study, ciRNAseq

## Abstract

**Background:**

Persistent infections with high-risk human papillomavirus (hrHPV) can cause cervical squamous intraepithelial lesions (SIL) that may progress to cancer. The cervicovaginal microbiome (CVM) correlates with SIL, but the temporal composition of the CVM after hrHPV infections has not been fully clarified.

**Methods:**

To determine the association between the CVM composition and infection outcome, we applied high-resolution microbiome profiling using the circular probe-based RNA sequencing technology on a longitudinal cohort of cervical smears obtained from 141 hrHPV DNA-positive women with normal cytology at first visit, of whom 51 were diagnosed by cytology with SIL six months later.

**Results:**

Here we show that women with a microbial community characterized by low diversity and high *Lactobacillus crispatus* abundance at both visits exhibit low risk to SIL development, while women with a microbial community characterized by high diversity and *Lactobacillus* depletion at first visit have a higher risk of developing SIL. At the level of individual species, we observed that a high abundance for *Gardnerella vaginalis* and *Atopobium vaginae* at both visits associate with SIL outcomes. These species together with *Dialister micraerophilus* showed a moderate discriminatory power for hrHPV infection progression.

**Conclusions:**

Our results suggest that the CVM can potentially be used as a biomarker for cervical disease and SIL development after hrHPV infection diagnosis with implications on cervical cancer prevention strategies and treatment of SIL.

**Supplementary Information:**

The online version contains supplementary material available at 10.1186/s12879-024-09455-1.

## Background

High-risk human papillomavirus (hrHPV) infections are associated with premalignant cervical lesions that may progress to cervical cancer [1]. Around 80% of all sexually active women will acquire an HPV infection during their lives and in most of the cases the virus is spontaneously cleared by the host immune system [2, 3]. In some women, however, hrHPV evades the immune response and the infection becomes persistent, promoting the development of squamous intraepithelial lesions (SIL) that eventually can progress to invasive cervical cancer [4, 5]. Despite increased use of HPV-vaccines to prevent hrHPV infection, cervical cancer represents a huge public health burden worldwide with over 500,000 diagnoses and over 300,000 deaths yearly [6]. Current screening programs include hrHPV DNA testing followed by cytology triage (Pap test). Overall, the clinical specificity of screening is low, resulting in high rates of overdiagnosis and overtreatment, and stratification of women who are at risk of hrHPV-induced cancer remains a challenge [7]. Thus, there is a remaining need to better understand the cervicovaginal ecosystem, and to discover and apply effective predictive biomarkers for early detection and treatment of SIL.

The cervicovaginal microbiome (CVM) is a promising candidate biomarker for cervical disease behavior [8, 9]. Changes in the composition of the cervicovaginal microbiota have been associated with bacterial vaginosis (BV), pre-term birth, and viral infections caused by HIV and hrHPV [10–12]. The CVM is structured in microbial community state types (CSTs) in which specific bacterial species dominate the microbiome or assemble in a diverse microbial population. In a healthy cervix, the CVM is characterized by dominance of *Lactobacillus* species such as *Lactobacillus crispatus* (CST I), while depletion of *Lactobacillus* species and colonization by *Gardnerella vaginalis*, *Atopobium vaginae*, and *Megasphaera genomosp type 1* (CST IV) is typical of dysbiosis [13, 14]. CST IV has been associated with hrHPV infection, viral persistence, viral-induced cervical lesions, and cervical cancer [15]. In contrast, *Lactobacillus*-dominated microbiomes have been correlated with hrHPV clearance and disease regression [15]. Most of these observations have been described in cross-sectional studies, and since the CVM is a highly dynamic ecological environment [16], a thorough understanding of how the microbiome changes in the course of hrHPV infection to SIL requires longitudinal microbiome profiling studies.

Evaluating the cervicovaginal microbiota’s role in health and disease mainly relies on 16S rRNA gene sequencing (16S RNA-seq) methods [17, 18]. Using 16S RNA-seq, Oh HY et al. described an association of microbial communities and species with SIL development in hrHPV-positive women [19]. Nevertheless, 16S RNA-seq yields only genus-resolution microbiome profiling for many taxa and provides limited species information due to the complexity of the variable regions (VRs) in the 16S rRNA gene [18]. Species-level microbiome profiling can be achieved by shotgun metagenomics or circular probe-based RNA sequencing (ciRNAseq) techniques [20]. Using shotgun metagenomics, Yan Q et al. found high abundance of *G. vaginalis* in HPV16-positive women [21]. However, shotgun metagenomics is relatively expensive, and it requires specialized resources for data analyses [22]. The ciRNAseq technology employs single-molecule molecular inversion probes (smMIPs) to target conserved DNA and RNA sequences in the 16S and 23S rRNA genes of microbial species within the CVM. ciRNAseq exhibits high specificity and sensitivity in identifying microbial species in mock community samples and women’s cervical smears [20]. Likewise, ciRNAseq provides improved taxonomic resolution compared to 16S RNA-seq, which is critical for the study of the CVM in hrHPV infections. Furthermore, by employing unique molecule identifiers (UMIs) the technique yields quantitative information irrespective of PCR-amplification bias [20]. Through ciRNAseq profiling of the CVM, our group has previously defined associations of the CVM with hrHPV-negative conditions and hrHPV-induced high-grade squamous intraepithelial lesions (HSIL) [20]. More recently, we identified subgroups of CSTs based on the abundance of bacterial species commonly overlooked by conventional sequencing methods due to their high level of sequence identity with other species [14, 23, 24]. Nonetheless, the temporal associations of these microbial communities and species with hrHPV infection outcomes are unknown.

In this longitudinal study, we investigate the composition of the CVM in a cohort of Dutch women participating in the population-based cervical cancer screening program, with proven hrHPV infection but normal cytology at baseline, who were diagnosed with SIL six-months later or did not develop cervical abnormalities. Our study aimed to evaluate the composition and temporal changes in the microbiome in relation to hrHPV progression in a 6-month period to identify potential early microbiome signatures associated cervical disease development after an hrHPV infection diagnosis. We show that an initial CST IV-A and high *G. vaginalis* or *A. vaginae* abundance associate with a progressive infection outcome at six-months, while *L. crispatus* dominance at both visits associates with non-progression. In addition to CSTs, we describe a combination of microbial species associated with hrHPV outcomes at both visits and relationships between bacteria occurring in the CVM. Our results suggest that the CVM is a valuable biomarker for hrHPV infection progression.

## Methods

### Study subjects and inclusion criteria

A total of 141 women participating in the Dutch population-based cervical cancer screening program and diagnosed with hrHPV infection and cytologically characterized as negative for intraepithelial lesion or malignancy (NILM) were included in the study. Women participating in the screening program were informed that residual material could be used for anonymous research and had the opportunity to opt out. Exclusion criteria included an hrHPV negative test result or diagnosis of squamous intraepithelial lesions (SIL) at first visit. Women without a follow-up sample were also excluded. Women were included irrespective of their ethnicity, parity, smoking habits, phase in their cycle, and use of contraception. At first visit (V1, time = 0 months) and second visit (V2, time = 6 months), 141 cervical smears in PreservCyt were included in the study and were processed and sequenced for microbiome profiling [20]. Five milliliters of each cervical cell suspension were centrifuged for 5 min at 2500 × g, and the pellet dissolved in 1 ml of TRIzol reagent (Thermo Scientific). RNA was isolated through standard procedures and dissolved in 20 μl nuclease-free water. We routinely processed a maximum of 2 μg of RNA for DNase treatment and cDNA generation, using SuperscriptII (Thermo). At V1, all women had sufficient RNA material for further processing. From the women with microbiome profiling at V2, a total of 83 cervical smears (58.8%) had sufficient material available for hrHPV DNA testing. The cytological follow-up outcomes at V2 were obtained for all participating women from the nationwide network and registry of histo- and cytopathology in the Netherlands (PALGA; Houten, The Netherlands). In this study we used liquid-based cytology (LBC) data according to the Bethesda coding system with categories NILM, low-grade squamous intraepithelial lesion (LSIL), and high-grade squamous intraepithelial lesion (HSIL).

### HrHPV identification and genotyping

HrHPV testing was performed with the Roche Cobas 4800 test, according to the manufacturer’s recommendations in the Department of Medical Microbiology at Radboudumc [25].

### CiRNAseq microbiome profiling and output analyses

High-resolution microbiome profiling was performed on ~ 50 ng of cDNA using the ciRNAseq technology [20, 26]. Probes (smMIPs) designed and selected to bind to framework regions flanking VRs in the 16S and 23S rRNA genes of microbial species [20] in the CVM were mixed with cDNA in a capture hybridization reaction and were circularized via a combined primer extension and ligation reaction. Circularized probes were subjected to PCR with barcoded Illumina primers. After purification of correct-size amplicons, quality control, and quantification [27], a 4 nM library was sequenced on the Illumina Nextseq500 platform (Illumina, San Diego, CA) at the Radboudumc sequencing facility to produce 2 × 151 bp paired-end reads.

Reads were mapped against reference regions of interest (ROIs) in our Cervicovaginal Microbiome Panel containing 321 microbial species[20] using the SeqNext module of JSI Sequence Pilot version 4.2.2 build 502 (JSI Medical Systems, Ettenheim, Germany). Our microbiome panel and ROIs were designed based on the most relevant species in the CVM and validated as previously described [20]. The settings for read processing were a minimum of 50% matching bases, a maximum of 15% mismatches, and a minimum of 50% consecutive bases without a mismatch between them; for read assignment, the threshold was a minimum of 95% of identical bases within the ROIs [20]. All identical PCR products were reduced to one consensus read (unique read counts, URC) using unique molecular identifiers (UMI), which consisted of a random 8-nucleotide sequence flanking the ligation probe in the smMIP and which is co-amplified during PCR. All FASTQs with identical (UMI) sequences therefore originate from the same circularized smMIP, allowing decomplexing of these sequences and making the assay insensitive of amplification bias. We set an arbitrary threshold of at least 1000 unique read counts (URC) from all smMIPs combined in an individual sample, below which we considered an output non-interpretable. Using a custom R script, microbial species were annotated when at least two reactive smMIPs for that species had URC. To define relative abundances, microbial species URC was divided by the total URC of all microbes annotated in the sample [20].

### Microbiome assessment and analyses

Hierarchical clustering (HC) and Partial least-squares discriminant analysis (PLSDA) were performed using ClustVis and MetaboAnalyst, respectively [28, 29]. The settings for HC were as follows: clustering distance for columns: Manhattan; clustering method: Ward. CSTs designation was performed through unsupervised clustering analyses [24]. CSTs were classified into five major groups (I to V) and the subgroups of CSTs I, III, and IV [14, 24] based on microbiome composition.

The predictive diagnostic potential of *A. vaginae*, *G. vaginalis*, *D. micraerophilus*, and *L. crispatus* for distinguishing non-progressive and progressive women at V1 were evaluated by a Random Forest analysis followed by receiver operating characteristic (ROC) curves of the bacterial species markers, and results were quantified by the area under the curve (AUC) using the randomForest [30] and pROC [31] R packages.

SankeyMATIC software was utilized to visualize the temporal changes in microbiomes. Pearson’s r partial correlations between microbial species were determined and generated with the ppcor R package [32]. The microbiome variation in the six-month period within a woman was obtained through a Jensen-Shannon distance (JSD) calculation in the philentropy R package [33]. JSD values give a measure of similarity between samples (i.e., by calculating the distance between samples) from the same woman. Low JSD values indicate similar microbial communities between samples, and conversely, large values indicate less similar communities.

### Statistical analysis

GraphPad Prism v9.4.0 (GraphPad Software, Inc., USA) was used to analyze datasets and determine the Shannon’s diversity indices and odds ratios. The statistical significance of differences was calculated using the Kruskal–Wallis test for multiple comparisons followed by a Benjamini–Hochberg test correction. Mann–Whitney U and Wilcoxon rank tests were employed for single and paired analyses, respectively. A McNemar’s test with a continuity correction was applied for matched-pairs analyses between both visits.

## Results

### Study design and hrHPV infection outcomes

**Fig. 1:**
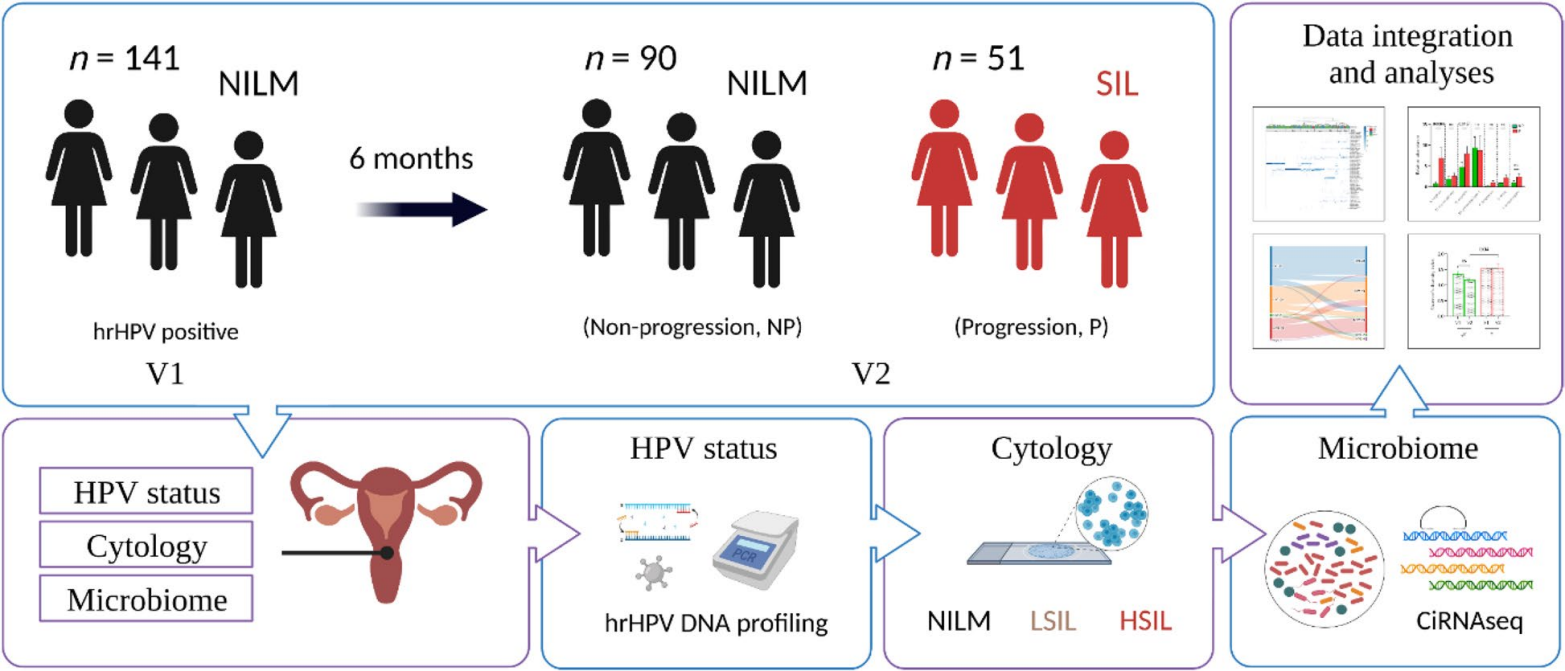
Study design. All 141 women entered the study at baseline with DNA confirmed hrHPV infection, no cervical abnormalities and CVM profiling. The results of follow-up cytology were assessed at 6 months to determine whether the individual had progressed to intraepithelial lesion or malignancy (LSIL, HSIL) or not (NILM). By 6 months, 90 women were confirmed for NILM, and 51 women had a LSIL (*n* = 41) or HSIL (*n* = 10) diagnosis. Experimental procedures, analysis, and integration were carried out as described in Methods

Cervical smears from 141 women with DNA-confirmed hrHPV infection and a cytological diagnosis of negative for intraepithelial lesion or malignancy (NILM) were profiled for CVM at first visit (V1). Of these, 90 women also had a diagnosis NILM at 6 months (63.8%) (non-progression group, NP) while 51 women (36.2%) were diagnosed with low-grade squamous intraepithelial lesions (LSIL) (41/51, 80.4%) or HSIL (10/51, 19.6%) (progression group, P) (Fig. 1).

### Early microbiome composition and hrHPV infection outcomes

**Fig. 2:**
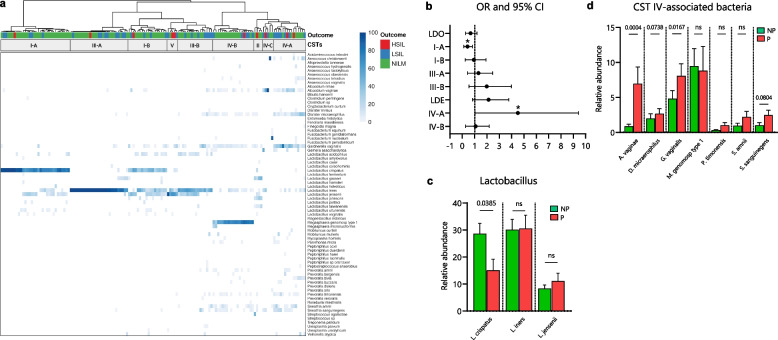
Early cervicovaginal microbiome composition is associated with hrHPV infection outcomes at six-months. **a** Cluster analysis of species-level profiling of the cervicovaginal microbiota at first collection visit (V1). Visualization of the distribution of hrHPV infection outcomes based on clusters show enrichment of NILM and SIL outcomes in specific communities. **b** Odd ratios (OR) and 95% confidence intervals comparing baseline CST groups (LDO: I, II, III, and V; LDE: IV) and individual CST subgroups for hrHPV infection progression at six-months. **c** Analyses of the relative abundances of *Lactobacillus* species in the CVM at V1 demonstrate association of *L. crispatus* with non-progression. **d** Analyses of the relative abundances of pathogenic anaerobes and the infection outcomes at six-months. OR in b were analyzed through a Fisher’s exact test, * *p* < 0.05. Differences in relative abundances were analyzed by using a Kruskal–Wallis test followed by the Benjamini–Hochberg test correction for multiple comparisons. *q* values are shown in **c** and **d** and error bars represent standard error of the mean ± s.e.m. *q* values < 0.10 are considered significant, ns = not significant. NP = non-progression group; P = progression group; LDO = *Lactobacillus*-dominated; LDE = *Lactobacillus*-depleted

Through unsupervised cluster analysis, we characterized the composition of the CVM in our longitudinal cohort at baseline (*n* = 141) and determined their association with cytological outcomes at six-months. Microbiomes clustered in CSTs dominated by *Lactobacillus* species: clusters I, III, and V (Fig. 2a, left clusters), and CSTs with a high diversity: clusters II and IV (Fig. 2a, right clusters, and Additional File 1: Supplementary Figure 1), including the subgroups of CSTs I (I-A, I-B), III (III-A, III-B) and IV (IV-A, IV-B, and IV-C) [24]. We did not observe a significant association between the overall baseline *Lactobacillus*-dominated (LDO, CSTs I, II, III, and V combined) and *Lactobacillus*-depleted (LDE, CSTs IV combined) microbiomes with hrHPV infection outcomes at V2 (Fig. 2a-b). Nevertheless, we see a clear trend where, of the CST types, CST I-A at baseline was most strongly associated with NILM at six-months (26/32, 81.2%, OR 0.32, 95% CI 0.12–0.82, *p* = 0.03, *q* = 0.15, Fisher’s exact test), while CST IV-A at baseline was most strongly associated with SIL outcomes at six-months (9/15, 60%, OR 3.07, 95% CI 1.03–9.40, *p* = 0.04, *q* = 0.16), however, the associations were only moderate when corrected for multiple testing (FDR < 0.2) (Fig. 2a-b).

To further explore the association of the CVM with progression to SIL, we examined it at level of individual bacterial species. We observed a significantly increased abundance of *L. crispatus* (*q* = 0.03, Kruskal Wallis test) in the NP group when compared to the P group (Fig. 2c). Moreover, we noticed that *A. vaginae* (*q* = 0.0004, Kruskal Wallis test), *G. vaginalis* (*q* = 0.01), *D. micraerophilus* (*q* = 0.07) and *S. sanguinegens* (*q* = 0.08), which are typical species found in CST IV, were more abundant in the P group than in the NP group (Fig. 2d).

### Dynamics of the microbiome and hrHPV infection outcomes

**Fig. 3:**
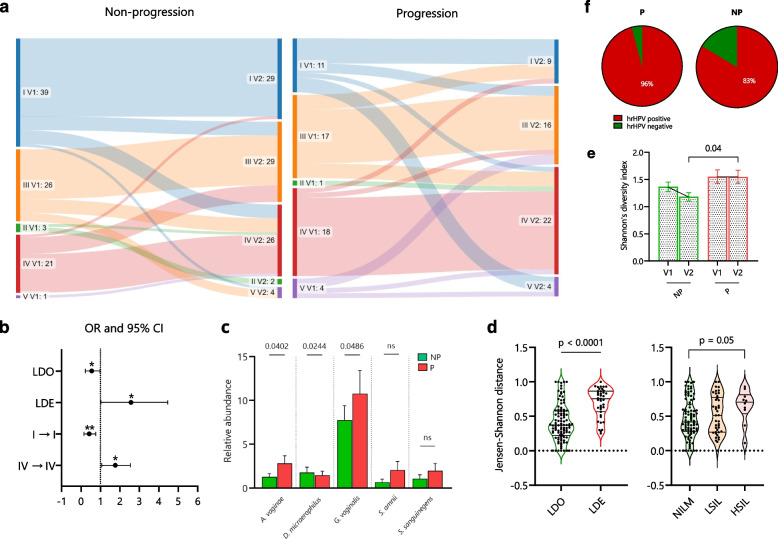
Dynamics of the microbiome and hrHPV infection outcomes. **a** The microbial shifts between both visits and groups. **b** Odd ratios (OR) and 95% confidence intervals comparing CST groups (LDO: I, II, III, and V; LDE: IV) at V2 and the six-months stability of CSTs I and IV with hrHPV infection outcomes. **c** Similarity of the CVM composition per microbial community and infection outcome through the Jensen-Shannon distance (JSD). **d** Comparison of relative abundances of the most abundant bacterial species associated with LDE microbiomes between NP and P groups. **e** Comparison of Shannon’s diversity indices for all microbiomes in NP and P groups at both visits. **f** Analysis on the hrHPV status in a subcohort of 83 women with hrHPV DNA testing at V2. OR in **b** were analyzed through a Fisher’s exact test, * *p* < 0.05, ** *p* < 0.01. Differences in relative abundances, JSD per outcomes, and Shannon indices per group, were analyzed by using a Kruskal–Wallis test followed by the Benjamini–Hochberg test correction for multiple comparisons. *q* values are shown in **d** and **e** and error bars represent standard error of the mean ± s.e.m. *q* values < 0.10 are considered significant, ns = not significant. Differences in JSD by *Lactobacillus* composition and paired Shannon indices were analyzed by using a Mann–Whitney U test and Wilcoxon matched-pairs test, respectively. NP = non-progression group; P = progression group; LDO = *Lactobacillus*-dominated; LDE = *Lactobacillus*-depleted

Six months after the initial diagnosis of hrHPV infection (V2), we again performed microbiome profiling on the cervical smears from all participating women (*n* = 141). We then established their CVM composition and examined the microbial changes between both visits and their association with hrHPV infection outcomes (Additional File 2: Supplementary Figure 2). Although we did not observe a significant association between CST subgroups and hrHPV infection outcomes at V2 like we observed at V1 (Fig. 2, Additional File 2: Supplementary Figure 2), we found that, LDE CSTs (IV) correlated with SIL (OR 2.21, 95% CI 1.02–4.45, *p* = 0.03, Fisher’s exact test), while LDO (I, II, III, and V) were associated with NILM (OR 0.45, 95% CI 0.22–0.97) (Fig. 3b).

We did not find a significant association between the CSTs II, III, IV, and V with microbiome stability in both groups (Fig. 3a). Nonetheless, compared to these CSTs in the NP group, CST I was significantly associated with a stable composition between both visits (*p* = 0.01, McNemar’s test) (Fig. 3a). Moreover, when analyzing the association between the six-months stability of CSTs with the hrHPV outcomes, we found that compared to other CSTs, stable CST I (OR 0.24, 95% CI 0.09–0.64, *p* = 0.003, Fisher’s exact test) and CST IV (OR 2.48, 95% CI 1.12–5.66, *p* = 0.03) were significantly associated with non-progression and progression, respectively (Fig. 3b).

Next, we examined the association of the CVM composition at V2 with the infection outcomes by comparing the relative abundances of pathogenic anaerobic species between both groups. Interestingly, we observed that the species *D. micraerophilus* (*q* = 0.02, Kruskal Wallis test) was more abundant in women with NILM than in women with SIL (Fig. 3c). Alternatively, we found that *A. vaginae* (*q* = 0.04), *G. vaginalis* (*q* = 0.04), *Prevotella bivia* (*q* = 0.008) and *Prevotella buccalis* (*q* = 0.008) were more abundant in the SIL group than in the NILM group (Fig. 3c and Additional File 3: Supplementary Figure 3).

To further evaluate the association of the temporal CVM composition with hrHPV infection outcomes, we calculated the Jensen-Shannon distance (JSD) [33] of the microbiome composition between both visits. A low JSD indicates a high similarity in microbial composition between the timepoints. We observed that women with LDO at baseline had a significantly more similar microbiome at V2 than women with LDE at baseline (*p* < 0.0001, Mann–Whitney U test) (Fig. 3d). Conversely, women with NILM outcomes had a more similar microbiome composition between both visits than those who developed HSIL (*p* = 0.05, *q* = 0.17, Kruskal Wallis test) (Fig. 3d).

We then assessed the Shannon’s diversity index and observed that women in both NP and P groups did not exhibit a significant change in microbial diversity from V1 to V2 (Wilcoxon matched-pairs test) (Fig. 3e). Nevertheless, women in the NP group had a significantly lower microbial diversity than the P group at V2 (*q* = 0.04, Kruskal Wallis test) (Fig. 3e). To test whether these microbial dynamics were associated with hrHPV persistence, we analyzed the hrHPV status for a subcohort of cervical smears with available hrHPV DNA testing at V2 (*n* = 83) and noticed that there were more hrHPV positive cervical smears in women with SIL (23/24, 96%) than women with NILM (49/59, 83%) (OR 4.69, 95% CI 0.68–53.04, *p* = 0.16, Fisher’s exact test) (Fig. 3f). Altogether, these findings demonstrate that prolonged *Lactobacillus* depletion, high microbial diversity, and increased abundance of CST IV-associated bacteria correlate with SIL. Conversely, a stable microbiome composition characterized by *Lactobacillus* dominance and low microbial diversity over a six-months period correlates with non-progression.

### Microbiome-based prediction of hrHPV infection outcomes

**Fig. 4:**
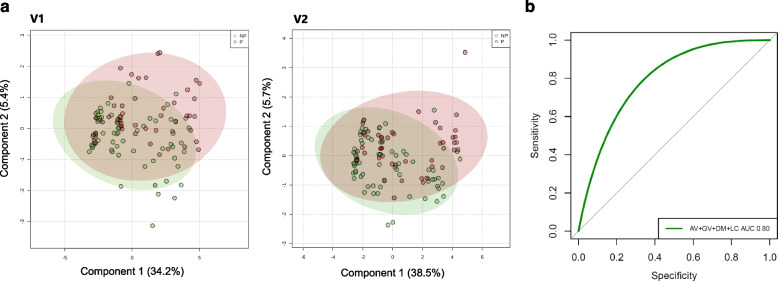
Cervicovaginal microbial species associated with hrHPV infection outcomes. **a** Partial least-squares discriminant analysis (PLSDA) of women’s CVM (*n* = 141) shows a similar separation between NP and P groups at both V1 and V2. **b** Receiver operating characteristics (ROC) curve and AUC of *A. vaginae* (AV), *G. vaginalis* (GV), *D. micraerophilus* (DM), and *L. crispatus* (LC) abundances in the microbiome from 141 hrHPV-positive women at V1 were calculated to generate a predictive model for the infection outcomes at six-months. These species combined exhibit a model with an AUC of 0.80 (95% CI 0.64–0.96)

Aside from estimating species that most significantly associate with the NP and P groups, we aimed to determine to what extent a combination of species associated with each group at both visits and whether such combination at V1 could be used to predict hrHPV infection outcomes at V2. To this purpose, we first performed a Partial least-squares discriminant analysis (PLSDA) with all microbiomes collected at V1 and V2 (*n* = 141) (Fig. 4a). We determined that *L. crispatus*, *A. vaginae*, *D. micraerophilus*, and *G. vaginalis* showed the strongest correlations with PLSDA Component 1 at both visits (Additional Files 4–5). Analyses of the Variable Importance in Projection (VIP) scores, a weighted sum of squares of the PLS loadings, showed consistent microbial species separating both groups in PLSDA C1 and the relative abundance associated with each group (Additional File 6: Supplementary Figure 4).

Next, we performed a Random Forest analysis and ROC to assess the performance of the early abundances in the CVM of the species *L. crispatus*, *A. vaginae*, *D. micraerophilus*, and *G. vaginalis*, which exhibited the strongest associations in our PLSDA (Fig. 4a), in predicting hrHPV outcomes at six-months. We found that these species together had a moderate discriminatory power at baseline for hrHPV infection progression at V2 with an AUC of 0.80 (95% CI 0.64–0.96) (Fig. 4b).

### Correlations among the cervicovaginal microbiota in hrHPV infections

**Fig. 5:**
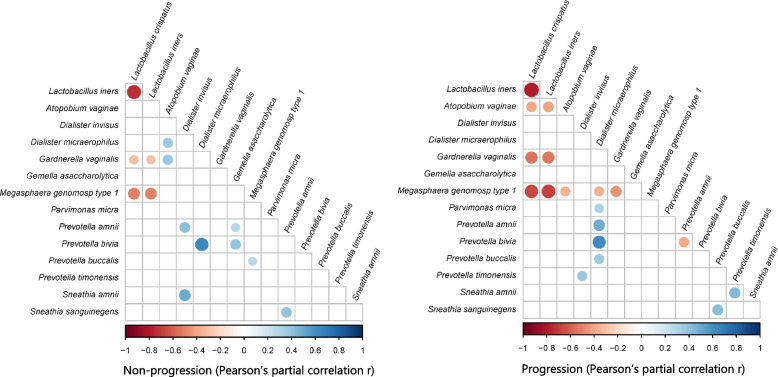
Interdependent relationships between the cervicovaginal microbiota in hrHPV infection. Pearson’s r partial correlations for multiple comparisons were estimated with the most abundant bacterial species in the microbiome of women in NP and P groups and integrating V1 and V2 datasets (*n* = 141). Color, size, and shade indicate the extent of positive and negative correlations. Correlation significance: *p* < 0.001

Lastly, to assess whether relationships between bacterial species were observed and persisted in non-progressive and progressive microbiomes, we performed Pearson’s partial r correlation analyses considering the most abundant species in both NP and P groups. We analyzed the microbial species abundances by integrating the two timepoints datasets to establish the correlations that persisted throughout both visits. In the NP group, there was a positive correlation between *A. vaginae* and *D. micraerophilus* and *G. vaginalis*. In the P group, there were inverse relationships between *Lactobacillus* and *A. vaginae*, between *M. genomosp type 1* and other pathogenic bacteria, and between *Prevotella* species (Fig. 5). Alternatively, we observed significant positive correlations between *D. micraerophilus* and *Prevotella* species and between *Prevotella* and *Sneathia* species in the P group (Fig. 5). In both groups, there was a negative correlation between *Lactobacillus* and *G. vaginalis* and *M. genomosp type 1* (Fig. 5). Likewise, both groups exhibited a significantly positive correlation between *D. micraerophilus* and *P. bivia* (Fig. 5). In conclusion, associations between *Lactobacillus* and pathogenic anaerobes, and between pathogenic anaerobes themselves, appear typical in the CVM during hrHPV infections.

## Discussion

The composition of the CVM not only correlates with hrHPV infections and cervical disease, but it may also predict the infection outcome. In this study, we observed that following hrHPV infection diagnosis, women with the microbial community IV-A [14, 24], characterized by *G. vaginalis* dominance and co-occurrence with *D. micraerophilus*, *A. vaginae*, *S. amnii*, and *S. sanguinegens* associate with progression to SIL six-months later. Pathogenic anaerobes such as *G. vaginalis* have been associated with viral persistence and cervical lesions [14, 34, 35]. Our findings are in line with previous longitudinal studies that established an association between bacterial vaginosis (BV) and the species *G. vaginalis* with cervical neoplastic lesions [34, 36]. Notably, we found that *A. vaginae* abundance at both visits is associated with infection progression and SIL at second visit. Increased *A. vaginae* abundance in the microbiome has been reported as a hallmark of SIL development [37, 38]. *A. vaginae* induces cytotoxic immune responses in cervicovaginal epithelial cells that reduces the protective mucosal layer, which might facilitate hrHPV persistence and integration into host cells [39, 40]. Thus, since these bacteria are highly abundant in hrHPV progressive infections and SIL, they could potentially be applied as biomarkers for cervical carcinogenesis. Moreover, these pathogenic species may represent promising targets for microbiome-based therapies against the development of cervical cancer.

Microbiomes abundant in *Lactobacillus* are associated with cervical health and their depletion results in cervical disorders [9, 37, 41, 42]. *Lactobacillus* species create a favorable microenvironment that allows sustained presence of lactate-producing bacteria and prevent outgrowth of harmful bacteria such as *G. vaginalis* [43, 44]. By this mechanism, *Lactobacillus* species may therefore prevent dysbiosis and persistent hrHPV infections [14, 45]. Additionally, microbial dominance by *L. crispatus* (CST I), *L. gasseri* (CST II), or *L. jensenii* (CST V) has been associated with hrHPV negative conditions, viral clearance, and regression of cervical lesions [46]. Although we did not assess this relationship in CSTs II and V due to the low prevalence of these CSTs in this study, these *Lactobacillus* species are known to stimulate a non-inflammatory state in the cervical epithelium, which facilitates effective immune responses against hrHPV infections and carcinogenesis [47]. Similarly, we described that LDO microbiomes have a more stable composition than LDE microbiomes. Since non-progressive women have microbial communities richer in *Lactobacillus* species than progressive women, this may explain the protection against hrHPV progression observed in our study [16, 48].

Aside from disease development, the microbiome dynamics also rely in the interactions of the cervicovaginal microbiota with the virus and the microenvironment [36, 49]. In our study, we describe that *Lactobacillus* species exhibit a strong negative relationship with CST IV-associated bacteria, which can be explained by the ecological conditions where they grow and their antimicrobial activities against pathogenic bacteria [50, 51]. Interestingly, *D. micraerophilus* showed strong positive associations with several *Prevotella* species in women with SIL outcomes, and in particular, a strong relationship with *P. bivia* was observed in both NP and P groups. *Prevotella* species often co-occur with *D. micraerophilus* and other pathogenic anaerobes in the CVM, and it is a clear example of how microbial species can associate with each other synergistically in the cervicovaginal environment [52]. *P. bivia* is an important source of ammonia and sialidase in the vaginal mucus and has been associated with cervical disease, which may explain its occurrence during infection [52]. Similarly, we observed that *P. timonensis* positively correlated with *S. amnii* in women with SIL outcomes (Fig. 5). *P. timonensis* interacts with vaginal dendritic cells, which are involved in mucosal inflammation [53, 54], and both species have been associated with viral persistence, slower regression of SIL, and cervical cancer [46, 55]. *Sneathia* and *Prevotella* species also express several homologous genes that are enriched in CST IV and that allow them to consume glycogen and mucins from cervical cells [56]. It could be hypothesized that *S. amnii* or *P. timonensis* may facilitate each other’s colonization, contributing to the risk of neoplastic lesions in hrHPV infection. This hypothesis is also consistent with the hrHPV downregulation of immune peptides that act as amino acid sources for *Lactobacillus* species, which leads to the growth of CST IV-associated bacteria in the CVM [57]. In general, the microbial dynamics during hrHPV infections remain interesting markers for infection behavior, but further studies are clearly needed to validate these associations in vitro and in vivo.

The strengths of the study are the use of the ciRNAseq technology for targeted sequencing of the microbiome and the application of longitudinal profiling in our study cohort. Potential limitations may include a short study period (six months) and a relatively small cohort size. Whereas the short study period may not be sufficient to capture the full spectrum of microbiome dynamics and their potential impact on disease progression, the cohort size may limit the statistical power of the study, making it challenging to detect subtle but potentially important associations. Furthermore, high-grade cases might have been missed during the visits due to the relatively low sensitivity of cervical cytology and the lack of histopathological data to further support the cytological analysis. Of note, although we included women with LSIL outcomes in our progression group, LSIL are considered non-progressive lesions [58], and therefore the microbiome associations described here should be considered carefully when investigating outcomes beyond six-months. Women with LSIL, however, developed these cervical abnormalities at V2 from diagnosed NILM at V1, which is defined as hrHPV infection progression. We were also unable to control for other cofounders, such as lifesytle, phase of the menstrual cycle, and antibiotic use during the study, which are known co-factors that impact on the CVM composition [59]. For instance, variations in hormone levels throughout the menstrual cycle can alter the CVM, and antibiotic use can disrupt microbial communities, potentially masking or exaggerating associations with disease outcomes. Therefore, a larger cohort and longitudinal clinical studies will be needed to validate our findings.

## Conclusions

In summary, we have shown how bacterial species, communities, dynamics, and relationships are relevant for assessing the role of the CVM in hrHPV carcinogenesis. This way, the CVM could be employed to support current cervical cancer prevention strategies and therapies against cervical lesions. Nonetheless, more studies and clinical trials are needed to properly assess and translate these findings in the clinic. Additionally, even though CSTs correlate with the infection outcome, their usefulness as biomarkers for cervical disease is not clear yet. Our in-depth analyses suggest species like *A. vaginae*, *G. vaginalis*, *L. crispatus*, and *D. micraerophilus* exhibiting strong associations with cervical conditions, and clustering species into CSTs does not necessarily result in better biomarkers than just examining the presence of a few species [60]. Further supervised analyses like Random Forest integrating host cell gene expression [61] to the microbiome data would be valuable to obtain a combination of biomarkers for disease progression, and such studies are on the way. Since ciRNAseq can provide bacterial information on DNA and RNA levels [20], while simultaneously perform transcriptome profiling [61], it could be applied to better understand the relationship of the microbiome with hrHPV infections.

### Supplementary Information

Additional file 1: Supplementary Figure 1. Microbial diversity of microbiomes at visit 1.

Additional file 2: Supplementary Figure 2. Composition of the microbiomes at visit 2.

Additional file 3: Supplementary Figure 3. Abundances of Prevotella species at visit 2.

Additional file 4. PLSDA Loadings at V1.

Additional file 5. PLSDA Loadings at V2.

Additional file 6: Supplementary Figure 4. Identification of relevant microbial species in the PLSDA.

Additional file 7. Sample Accession Numbers in NCBI database. Sample ID, hrHPV status, and accession numbers per visit.

## Data Availability

The sequence read data generated in this study are available at NCBI in the Sequencing Read Archive, projects PRJNA856437 (174 files) [62] and PRJNA888791 (108 files) [63] (Sample Accession Numbers are shown in Additional File 7).

## Abbreviations

AUC: Area under the curve
BV: Bacterial vaginosis
CiRNAseq: Circular probe-based RNA sequencing
CSTs: Community state types
CVM: Cervicovaginal microbiome
HC: Hierarchical clustering
hrHPV: High-risk human papillomavirus
HSIL: High-grade squamous intraepithelial lesions
JSD: Jensen-Shannon distance
LDO: *Lactobacillus*-Dominated
LDE: *Lactobacillus*-Depleted
LSIL: Low-grade squamous intraepithelial lesions
NILM: Negative for intraepithelial lesions or malignancy
NP: Non-progression
P: Progression
PLSDA: Partial least squares discriminant analysis
ROC: Receiver operating characteristic
SIL: Squamous intraepithelial lesions
URC: Unique read counts
