# Supplementary material for: Temporal composition of the cervicovaginal microbiome associates with hrHPV infection outcomes in a longitudinal study
Source: BMC Infect Dis. 2024 Jun 3;24:552. doi: 10.1186/s12879-024-09455-1 (PMC11145797; doi:10.1186/s12879-024-09455-1)
Supplement: Supplementary file 1 — Additional file 1: Supplementary Figure 1. Microbial diversity of microbiomes at visit 1. [file 12879_2024_9455_MOESM1_ESM.pdf]

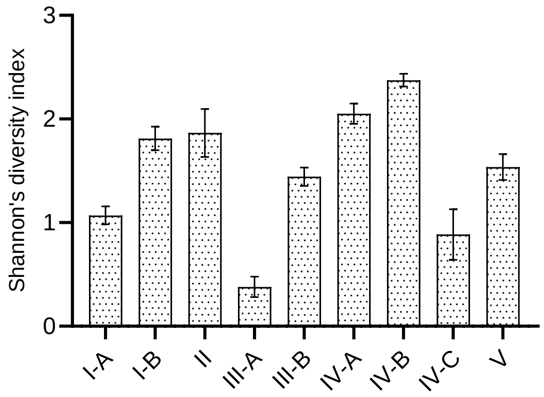

Supplementary Figure 1. Microbial diversity of microbiomes at V1.

Analysis of alpha-diversity of the CVM of all participating women at baseline per CSTs as evaluated by Shannon's index (*n* = 141). Error bars represent standard error of the mean ± s.e.m.
