# Supplementary material for: Temporal composition of the cervicovaginal microbiome associates with hrHPV infection outcomes in a longitudinal study
Source: BMC Infect Dis. 2024 Jun 3;24:552. doi: 10.1186/s12879-024-09455-1 (PMC11145797; doi:10.1186/s12879-024-09455-1)
Supplement: Supplementary file 2 — Additional file 2: Supplementary Figure 2. Composition of the microbiomes at visit 2. [file 12879_2024_9455_MOESM2_ESM.pdf]

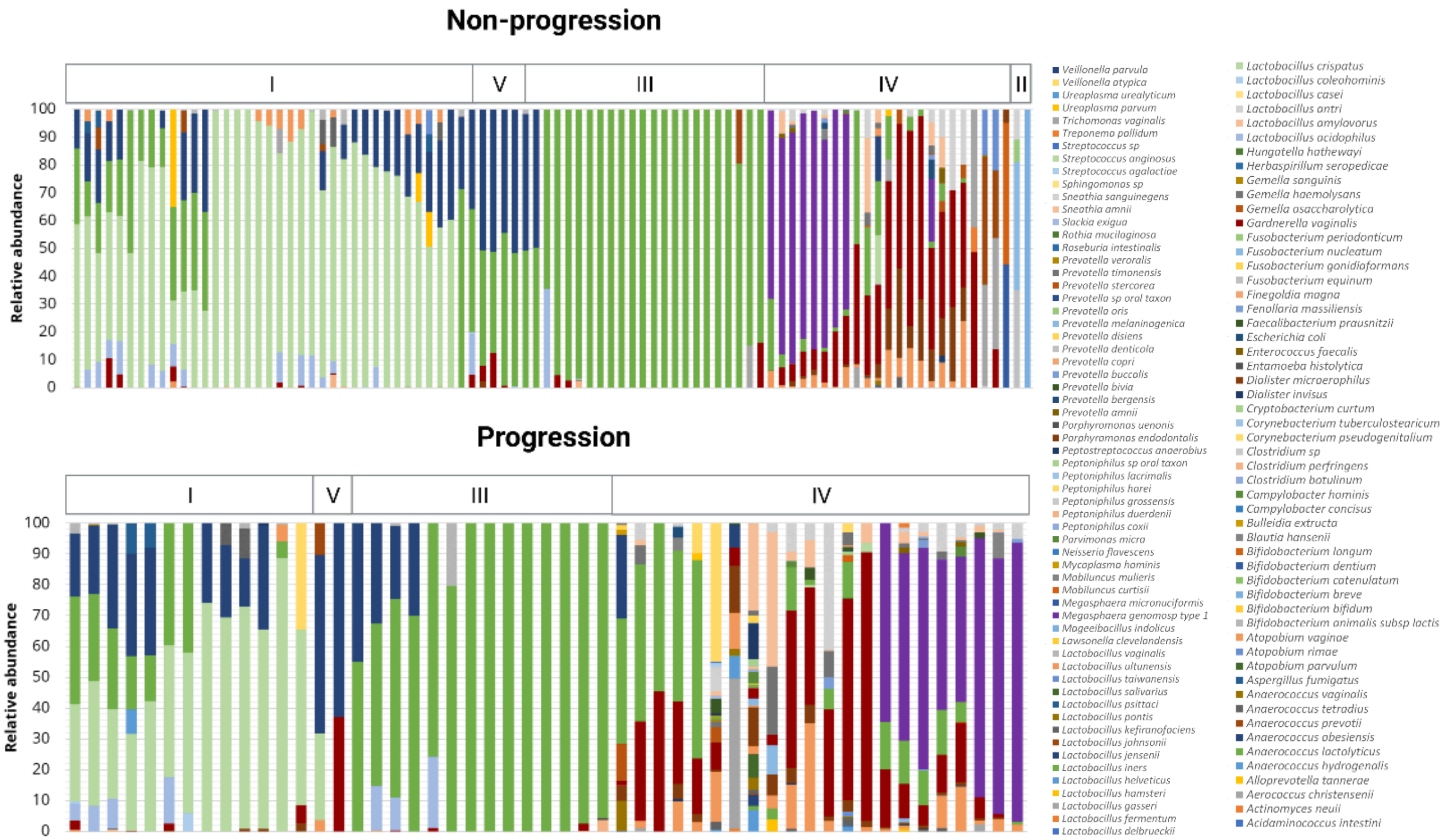

Supplementary Figure 2. Composition of the microbiomes at V2.

The cervicovaginal microbiota composition at second collection visit (V2) structures in CSTs based on unsupervised cluster analysis of the microbiomes and is displayed in graph bars (n = 141). The NP group is enriched for CSTs I, II, III, and V, while the P group is enriched for CST IV.
