# Supplementary material for: Temporal composition of the cervicovaginal microbiome associates with hrHPV infection outcomes in a longitudinal study
Source: BMC Infect Dis. 2024 Jun 3;24:552. doi: 10.1186/s12879-024-09455-1 (PMC11145797; doi:10.1186/s12879-024-09455-1)
Supplement: Supplementary file 3 — Additional file 3: Supplementary Figure 3. Abundances of Prevotella species at visit 2. [file 12879_2024_9455_MOESM3_ESM.pdf]

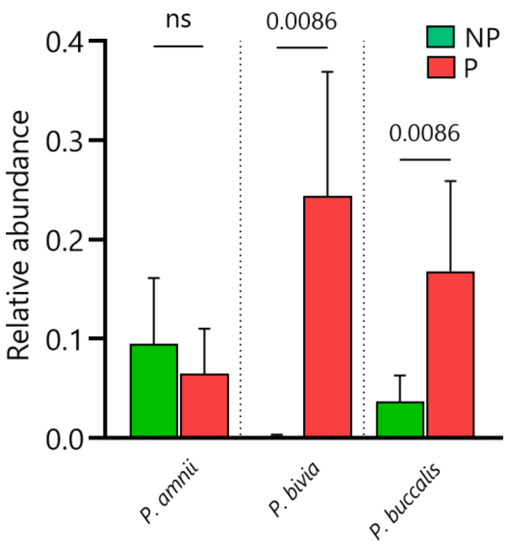

Supplementary Figure 3. Abundances of *Prevotella* species at V2.

Analyses of the relative abundances of *Prevotella* species in the CVM of all participating women at second visit (*n* = 141, V2). There is a significantly higher abundance for *P. bivia* and *P. buccalis* in the P group than in the NP group. Differences in relative abundances were analyzed by using a Kruskal-Wallis test followed by the Benjamini-Hochberg test correction for multiple comparisons. *q* values are displayed, and error bars represent standard error of the mean ± s.e.m. *q* values < 0.10 are considered significant, ns = not significant. NP = non-progression group; P = progression group.
