# Supplementary material for: Temporal composition of the cervicovaginal microbiome associates with hrHPV infection outcomes in a longitudinal study
Source: BMC Infect Dis. 2024 Jun 3;24:552. doi: 10.1186/s12879-024-09455-1 (PMC11145797; doi:10.1186/s12879-024-09455-1)
Supplement: Supplementary file 6 — Additional file 6: Supplementary Figure 4. Identification of relevant microbial species in the PLSDA. [file 12879_2024_9455_MOESM6_ESM.pdf]

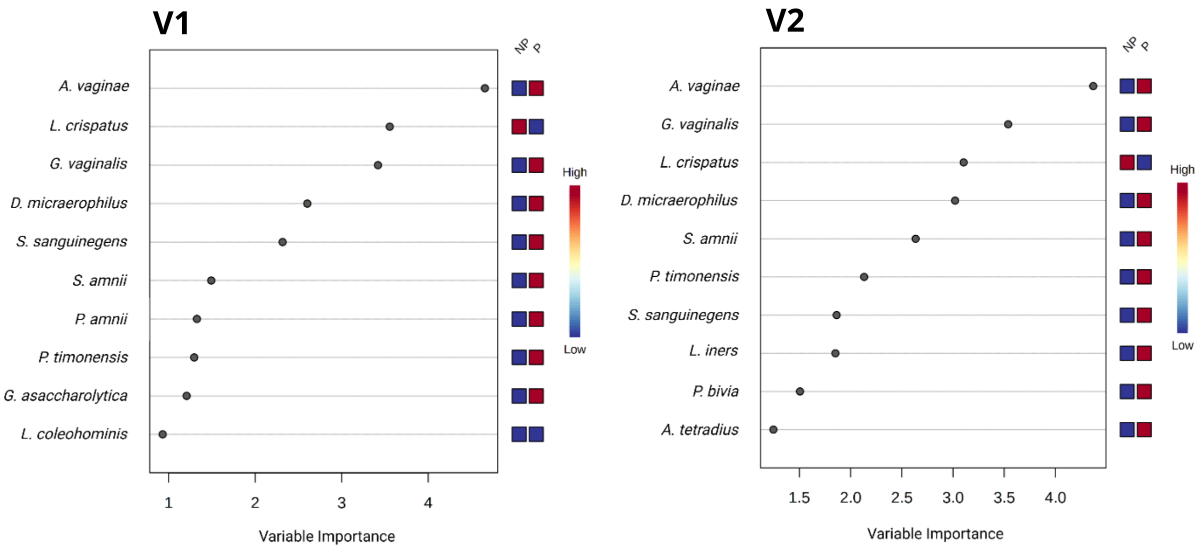


**Supplementary Figure 4. Identification of relevant microbial species in the PLSDA.**

Two component partial least-squares discriminant analysis (PLS-DA) was used to identify the top 10 microbial species that are the most contributory variables in PLSDA C1 for class discrimination between the non-progressive women and progressive women at both collection visits. The index values of the Variable Importance in Projection (VIP) from the PLS-DA component 1 show the species with VIP scores over one. The higher value of the VIP scores, the more important for that specie. VIP scores are a weighted sum of squares of the PLS loadings. The relative abundance of microbial species is indicated by a colored scale from blue to red representing the low and high, respectively. NP = non-progression group; P = progression group.
